# Older age increases the risk of revision and perioperative complications after high tibial osteotomy for unicompartmental knee osteoarthritis

**DOI:** 10.1038/s41598-021-03259-y

**Published:** 2021-12-21

**Authors:** Sun-Ho Lee, Hyoung-Yeon Seo, Hae-Rim Kim, Eun-Kyoo Song, Jong-Keun Seon

**Affiliations:** 1 Department of Orthopedic Surgery, Chonnam National University Medical School and Hospital, 322 Seoyang-ro, Hwasun-eup, Hwasun-gun, Jeollanam-do Republic of Korea; 2 College of Natural Science, School of Statistics, University of Seoul, Seoul, Republic of Korea

**Keywords:** Diseases, Medical research

## Abstract

Among various patient risk factors affecting survival after high tibial osteotomy (HTO), the ideal age limit for HTO is unclear. This study was performed to evaluate the effect of age on survival rate and complications after HTO for medial unicompartmental osteoarthritis. Among of 61,145 HTO patients from Korean National Health Insurance database, 41,112 patients underwent the procedure before the age of 60 years (Group A), 13,895 patients between the age of 60 and 65 years (Group B), and 6138 patients after the age of 65 years (Group C). We compared the survival rate in person-years among the three groups from the date of primary surgery until subsequent total knee arthroplasty. Perioperative complications were also recorded. The adjusted hazard ratio (HR) were calculated using the multivariable Cox proportional hazard regression model, adjusting for the potential confounders: age, sex, type of medical insurance, region of residence, hospital type, comorbidities, and Charlson comorbidity index score. The total number of HTO increased 6.5-fold, especially in patients aged > 65 years (by 8.2-fold) from 2008 to 2018. The overall revision rate was 4.2% in Group A, 6.4% in Group B, and 7.3% in Group C. The 5- and 10-year revision rate was significantly lower in Group A (p < 0.001), but no difference between Groups B and C. After adjusting for potential confounders, multivariable regression analysis revealed that revision rate was significantly lower in Group A than Group B (HR: 0.57; p < 0.0001), but no difference between Groups B and C. The incidence of complications was also significantly lower in Group A than in other groups. The inferior survival rate and more perioperative complications after HTO was found in old patients (aged ≥ 60 years) than in young patients. Therefore, the patient age is one of the predicting factors for a high risk of failure after HTO.

## Introduction

High tibial osteotomy (HTO) is widely used to correct varus malalignment in the management of medial unicompartmental osteoarthritis (OA), especially in young active patients^1–3^. The procedure can relieve knee pain and preserve the native knee joint. Many studies have found that HTO confers excellent long-term survival rates and postoperative results in relatively young patients^3,4^. Moreover, with improvements in surgical methods and advances in implant design, the range of indications for HTO is expanding^5–7^.

As more HTO procedures are being performed, many studies have investigated the risk factors, especially patient variables, affecting failure after surgery^8–21^. Although Brinkmean et al. defined the age of ideal patients for HTO as 40–60 years^3^, the age indications for HTO is expanded due to improvements in surgical methods and implant design. However, there is no consensus regarding ideal age limit for HTO. Some studies have reported that age does not influence clinical outcome or survival after HTO^17,22^, while other studies^12,14,15,18,23–25^ have shown better clinical outcomes in younger patients than in older patients. Jin et al.^14^ and Trieb et al.^26^ reported higher failure rates after HTO in patients aged > 65 years than in patients aged < 65 years. However, most of these studies involved a relatively small number of patients. Moreover, no large comparative series studies have used national registry data to investigate the effect of age on survival after HTO.

To the best of our knowledge, this long-term, follow-up-based, nationwide cohort study with adequate power is the most extensive study performed thus far to evaluate the effect of age on the survival rate and complications after HTO for medial unicompartmental osteoarthritis (OA). We hypothesized that younger patients show better survival and fewer complications after HTO than older patients.

## Materials and methods

This study was approved by the Institutional Review Board (IRB) of Chonnam National University Hwasun Hospital. The review board waived the requirement for patient consent because the retrospective nature of the study.

### Data sources

**Figure 1:**
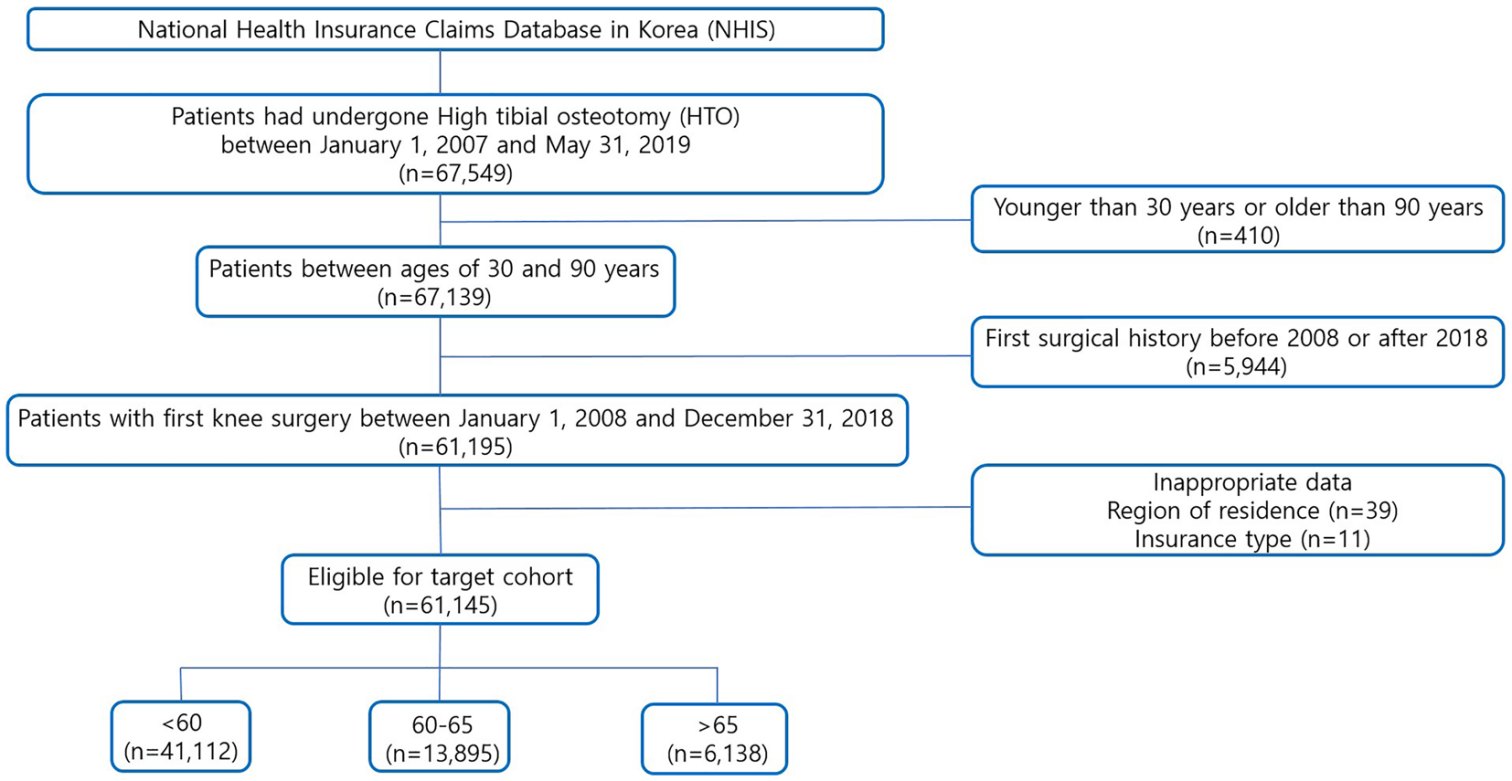
Flowsheet for Eligibility.

The study population included all patients who underwent HTO in Korea between January 1, 2007 and May 31, 2019. To allow a washout period of 1 year, the index date was set as January 2008 (Fig. 1).

We identified the target cohort by searching for HTO using the surgical procedure code to ensure that cases counted in only one of the registers were included. This retrospective nationwide cohort study used the claims database of the National Health Insurance Service (NHIS; Seoul, South Korea), which covers almost 99% of entire Korean population. Diagnosis was based on the International Classification of Diseases, 10th revision (ICD-10), and the procedure code was based on the Electronic Data Interchange (EDI). The NHIS database of South Korea provides data on the individual identification codes, and the data linked to each code provides information on patient age, sex, diagnosis, hospitalization records, surgical records, medication prescriptions, and local hospital information.

### Data collection

The study population included all patients aged 30–90 years who underwent HTO (EDI: N0304) as a primary procedure. Each patient’s first additional procedure throughout the study period was also recorded. Patients who had undergone orthopedic surgery during the preceding year (washout period) were excluded to eliminate the influence of previous surgical history. All diagnosis and treatment codes were defined and searched in accordance with the ICD-10 and EDI codes. The first objective of the study was to confirm age-related revision rates; the second objective was to analyze the incidence of unwanted surgery-related complications according to age. We compared survival rates and complications according to age (< 60 years, 60–65 years, > 65 years) by analyzing the conversion to unicompartmental knee arthroplasty (UKA) or total knee arthroplasty (TKA) and evaluating perioperative medical complications. This was approved by the Institutional Review Board of our hospital. The review board waived the requirement for patient consent because the retrospective nature of the study.

### Statistical analysis

The following baseline characteristics of patients treated with HTO were collected: age, sex, type of insurance, region of residence, hospital type, and comorbidities. These were summarized using descriptive statistics, including proportion, mean, and standard deviation. Differences in continuous variables among age groups (< 60, 60–65, and > 65 years) were evaluated using the Wilcoxon rank-sum test, while categorical variables were compared using the chi-square test. The balance of covariates among groups was evaluated using standardized mean difference (SMD). An SMD of < 0.1 indicated a negligible difference between the groups. In each group, we calculated the person-years from the date of primary surgery until subsequent TKA or UKA. We also recorded perioperative complications. Time to revision surgery was calculated using Kaplan–Meier survival analysis. The adjusted hazard ratio (HR) and 95% confidence interval (CI) were calculated using a multivariable Cox proportional hazard regression model, adjusting for potential confounders such as age, sex, type of medical insurance, region of residence, hospital type, comorbidities, and Charlson comorbidity index (CCI) score. Patients aged 60–65 years were placed in a reference group, while those aged < 60 years were compared with those aged > 65 years. Data were analyzed using R software (version 3.4.1; R Foundation for Statistical Computing) and SAS Enterprise software (version 6.1; SAS Institute).

### Ethics approval

Institutional Review Board (IRB) of Chonnam National University Hwasun Hospital approved this study.

### Informed consent

For this type of study, formal consent is not required.

## Results

A total of 67,549 patients who underwent HTO during the 11.5-year period from January 2007 to March 2019 were identified. Patients aged < 30 years and > 90 years (n = 410) and those with inappropriate data (n = 50) were excluded. The final target cohort included 61,145 patients who had undergone HTO. Of the 61,145 patients, 41,112 patients underwent the procedure before the age of 60 years (Group A), 13,895 patients underwent the procedure between the age of 60 and 65 years (Group B), and 6138 patients underwent the procedure after the age of 65 years (Group C; Fig. 1). The mean age was 52.6 years in Group A, 62.1 years in Group B, and 70.1 years in Group C (p < 0.001). Group A had a higher proportion of male patients (73.4%) than Groups B and C, which had approximately 77% female patients. Underlying diseases (hypertension, hyperlipidemia, peripheral vascular disease, diabetes mellitus, and depression) were more common in Groups B and C than in Group A (Table 1).

**Table 1:** Patient baseline characteristics of high tibial osteotomy by age.

	HTO				
	< 60	60–65	> 65	P-value	SMD
	(N = 41,112)	(N = 13,895)	(N = 6138)		
Age (mean (sd))	52.62 (5.38)	62.10 (1.66)	70.13 (4.24)	< 0.001	2.829
**Sex (%)**				< 0.001	0.052
Female	10,948 (26.6)	3230 (23.2)	1431 (23.3)		
Male	30,164 (73.4)	10,665 (76.8)	4707 (76.7)		
Hypertension (%)	15,858 (38.6)	7465 (53.7)	3981 (64.9)	< 0.001	0.360
Hyperlipidemia (%)	18,933 (46.1)	8518 (61.3)	3677 (59.9)	< 0.001	0.206
Peripheral vascular disease (%)	7898 (19.2)	3915 (28.2)	2010 (32.7)	< 0.001	0.208
Diabetes_without complication (%)	8120 (19.8)	4096 (29.5)	2230 (36.3)	< 0.001	0.250
Diabetes_with complication (%)	2898 (7.0)	1579 (11.4)	930 (15.2)	< 0.001	0.174
Depression (%)	6063 (14.7)	2631 (18.9)	1277 (20.8)	< 0.001	0.106
Dementia (%)	252 (0.6)	248 (1.8)	373 (6.1)	< 0.001	0.213
**Type of insurance (%)**				0.68	0.083
Health insurance	39,522 (96.1)	13,563 (97.6)	5852 (95.3)		
Medical benefits	1590 (3.9)	332 (2.4)	286 (4.7)		
**City of residence (%)**				< 0.001	0.085
Over 10milion	11,345 (27.6)	4099 (29.5)	1581 (25.8)		
Over 1milion	11,603 (28.2)	3736 (26.9)	1550 (25.3)		
Others	18,164 (44.2)	6060 (43.6)	3007 (49.0)		
**Type of hospital (%)**				< 0.001	0.091
Teaching hospital	4734 (11.5)	1277 (9.2)	645 (10.5)		
General hospital	7369 (17.9)	2336 (16.8)	1229 (20.0)		
Independent hospital	27,353 (66.5)	9603 (69.1)	3956 (64.5)		
Private clinic	1656 (4.0)	679 (4.9)	308 (5.0)		
**CCI (%)**				< 0.001	0.329
0	4617 (11.2)	733 (5.3)	271 (4.4)		
1	8530 (20.7)	1760 (12.7)	663 (10.8)		
2	9465 (23.0)	2835 (20.4)	1069 (17.4)		
≥ 3	18,500 (45.0)	8567 (61.7)	4135 (67.4)		
**Year (%)**				< 0.001	0.183
2008	1143 (2.8)	236 (1.7)	128 (2.1)		
2009	1302 (3.2)	236 (1.7)	164 (2.7)		
2010	1591 (3.9)	290 (2.1)	172 (2.8)		
2011	2403 (5.8)	577 (4.2)	308 (5.0)		
2012	3192 (7.8)	807 (5.8)	396 (6.5)		
2013	4125 (10.0)	1204 (8.7)	579 (9.4)		
2014	4253 (10.3)	1274 (9.2)	575 (9.4)		
2015	5153 (12.5)	1626 (11.7)	750 (12.2)		
2016	6039 (14.7)	2225 (16.0)	1004 (16.4)		
2017	5952 (14.5)	2580 (18.6)	1008 (16.4)		
2018	5959 (14.5)	2840 (20.4)	1054 (17.2)		

*HTO* high tibial osteotomy, *SMD* standardised mean difference, *CCI* Charlson comorbidity index.

*CCI: Myocardial infarction, Congestive heart failure, Peripheral vascular disease, Cerebrovascular disease, Dementia, Chronic pulmonary disease, Connective tissue disease, Peptic ulcer disease, Mild liver disease, Moderate or severe liver disease (3), Diabetes without complications, Diabetes with complications (2), Paraplegia and hemiplegia (2), Renal disease (2), Cancer (2), Metastatic carcinoma (6), AIDS/HIV (6).

Type of medical insurance did not differ among the three groups, but there was a difference in city of residence and hospital type. The CCI score was significantly higher in Groups B and C than in Group A. The proportion of patients in Group B or C significantly increased over the 10 years (Table 1). The number of HTO procedures increased by 6.5-fold, from 1507 in 2008 to 9853 in 2018, while the number of patients undergoing the procedure after the age of 65 years increased by 8.2-fold (Table 1).

**Figure 2:**
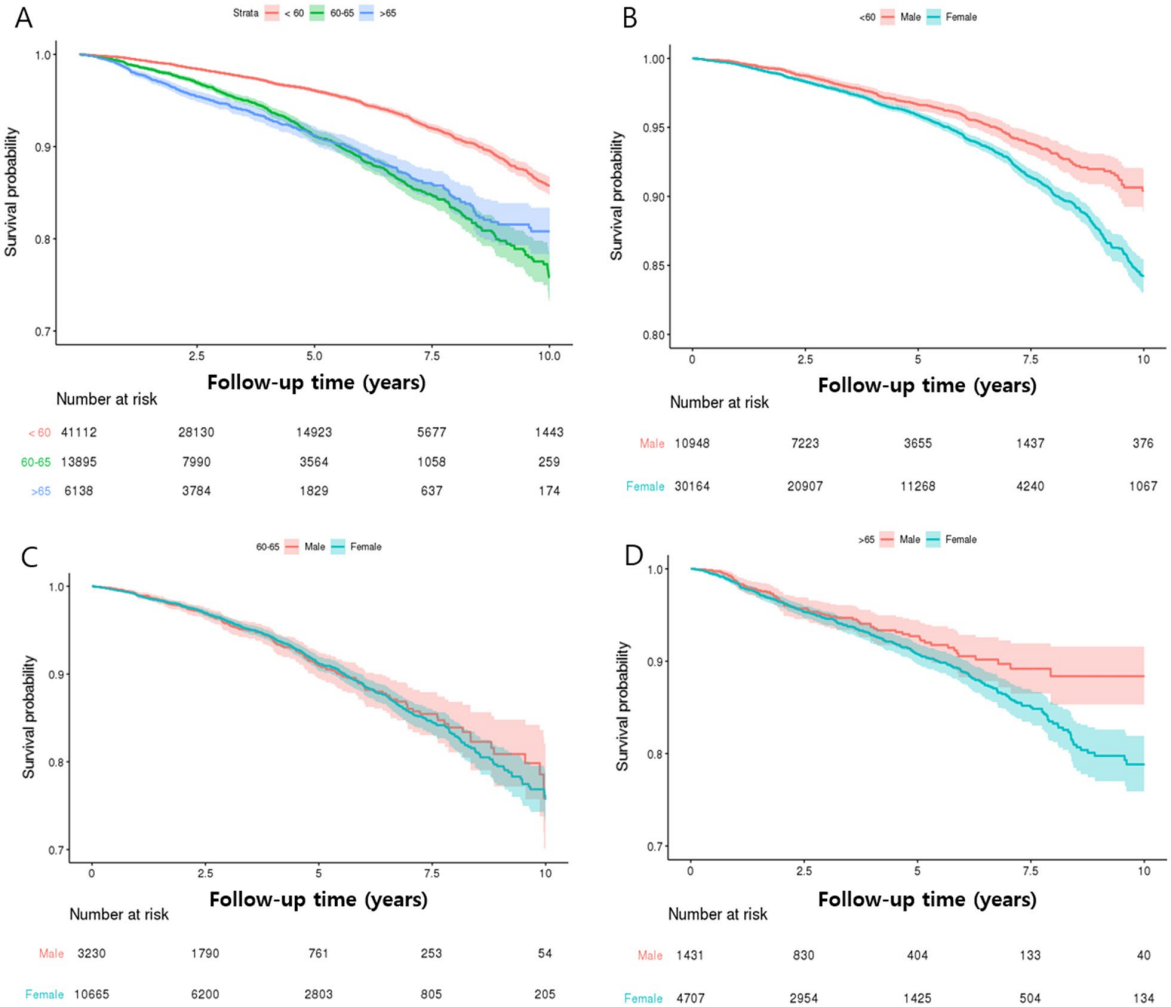
Kaplan–Meier survivorship curve: 11.5-year survivorship among different age groups. (**A**) Total patients, (**B**) Gender survival curve under 60 years old, (**C**) Gender survival curve between 60 and 65, (**D**) Gender survival curve over 65.

The overall revision rate was 4.2% (CI: 95.1–95.9) in Group A, 6.4% (CI: 94.9–96.5) in Group B, and 7.3% (CI: 94.9–96.5) in Group C. There was a significant difference in the revision rate among the three groups after 5 and 10 years, with Group A showing a significantly lower revision rate than other groups (p < 0.001); however, there was no difference between Groups B and C (p > 0.05; Table 2; Fig. 2A). Moreover, male patients had better survival in all age groups after 5 and 10 years (Fig. 2B–D).

**Table 2:** Cumulative revision incidence for stratified age subgroups.

		Under 60 (n = 41,112)			60–65 (n = 13,895)			Over 65 (n = 6138)		
		N	%	1000 PY	N	%	1000 PY	N	%	1000 PY
Revision	event	1707	(4.15%)	9.71	885	(6.37%)	18.01	449	(7.32%)	19.05
	days	1573.39	± 991.74		1314.49	± 879.28		1114.07	± 827.48	
Revision (5 year)	event	1051	(2.56%)	7.40	663	(4.77%)	15.73	355	(5.78%)	18.10
	days	905.41	± 486.38		906.19	± 516.35		763.86	± 481.74	
Revision (10 year)	event	1668	(4.06%)	9.54	875	(6.30%)	17.87	449	(7.32%)	19.13
	days	1520.07	± 938.96		1285.83	± 841.96		1114.07	± 827.48	

*PY person year.

The number of 10-year revisions per 1000 person-years was 9.5 in Group A, 17.8 in Group B, and 19.1 in Group C (Table 2). Group A had a significantly lower revision rate than Group B (HR: 0.5, 95% CI: 0.46–0.54; p < 0.001); the HR was 0.46 after 5 years and 0.49 after 10 years (Table 3). However, there was no significant difference in the revision rate between Groups B and C. After adjusting for potential confounders, such as age, sex, type of medical insurance type, region of residence, type of hospital, comorbidities, and CCI score, multivariable regression analysis showed that the revision rate was significantly lower in Group A than in Group B (HR: 0.57, 0.53–0.62, p < 0.0001); the HR was 0.53 after 5 years and 0.57 after 10 years (Table 3). However, there was no significant difference in the revision rate between Groups B and C (HR: 0.96, 0.86–1.08; p = 0.53).

**Table 3:** COX proportional hazard survival analysis for risk of revision.

	60–65 (n = 13,895)	Under 60 (n = 41,112)							Over 65 (n = 6138)					
		Crude			Adjusted			Crude				Adjusted		
		HR	95% CI	P-value	HR	95% CI	P-value	HR		95% CI	P-value	HR	95% CI	P-value
Revision	Reference	0.50	(0.46,0.54)	< 0.0001	0.57	(0.53,0.62)	< 0.0001	1.02		(0.91,1.14)	0.78	0.96	(0.86,1.08)	0.53
Revision(5 year)	Reference	0.46	(0.41,0.50)	< 0.0001	0.53	(0.48,0.58)	< 0.0001	1.14		(0.99,1.29)	0.051	1.07	(0.94,1.21)	0.33
Revision(10 year)	Reference	0.49	(0.46,0.54)	< 0.0001	0.57	(0.52,0.62)	< 0.0001	1.03		(0.92,1.15)	0.61	0.98	(0.87,1.09)	0.68

*HR* hazard ratio, *CI* confidence interval, reference: age of 60–65.

*Adjusted variable: age, sex, comorbidities, type of insurance, type of hospital, region of residence, CCI.

In terms of complications, cerebrovascular accident (CVA; HR: 0.68), myocardial infarction (MI; HR: 0.65), and delirium (HR: 0.31) were significantly lower in Group A than in Group B after adjusting for confounders. Moreover, Group C showed a significantly higher rate of pulmonary thromboembolism (PTE; HR: 1.97), CVA (HR: 1.55), MI (HR: 1.52), acute respiratory failure (ARF; HR: 2.24), and delirium (HR 2.57) than Group B (Tables 4, 5). Surgical site infection was also significantly higher in Group C than in Group B (HR: 1.61, p = 0.001, Table 5).

**Table 4:** Cumulative incidence of adverse outcomes for stratified age subgroups.

	Under 60 (n = 41,112)			60–65 (n = 13,895)			Over 65 (n = 6138)		
	N	%	1000 PY	N	%	1000 PY	N	%	1000 PY
**Deep vein thromboembolism**									
Event	188	(0.46%)	1.07	73	(0.53%)	1.49	47	(0.77%)	1.99
Days	777.49	± 986.27		664.55	± 816.72		608.77	± 766.87	
**Pulmonary thromboembolism**									
Event	78	(0.19%)	0.44	36	(0.26%)	0.73	35	(0.57%)	1.48
Days	621.59	± 857.94		499.31	± 664.61		837.69	± 870.90	
**Cerebrovascular disease**									
Event	4247	(10.33%)	24.15	2079	(14.96%)	42.32	1520	(24.76%)	64.49
Days	908.56	± 877.75		714.58	± 780.58		702.81	± 774.83	
**Myocardial infarction**									
Event	253	(0.62%)	1.44	133	(0.96%)	2.71	105	(1.71%)	4.45
Days	1039.91	± 971.16		741.02	± 855.08		702.65	± 871.45	
**Acute renal failure**									
Event	180	(0.44%)	1.02	64	(0.46%)	1.30	81	(1.32%)	3.44
Days	1177.04	± 1002.03		820.64	± 870.51		1220.04	± 914.74	
**Postoperative Delirium**									
Event	18	(0.04%)	0.10	19	(0.14%)	0.39	30	(0.49%)	1.27
Days	788.00	± 733.98		1524.16	± 940.90		752.73	± 821.77	
**Surgical site infection**									
Event	311	(0.76%)	1.77	107	(0.77%)	2.18	84	(1.37%)	3.56
Days	518.28	± 764.08		582.71	± 819.77		384.49	± 588.73	

*PY person year.

**Table 5:** COX proportional hazard analysis for risk of perioperative complications.

60–65 (n = 13,895)	Under 60 (n = 41,112)						Over 65 (n = 6138)					
	Crude			Adjusted			Crude			Adjusted		
	HR	95% CI	P-value	HR	95% CI	P-value	HR	95% CI	P-value	HR	95% CI	P-value
**Deep vein thromboembolism**												
Reference	0.78	(0.59,1.02)	0.07	0.87	(0.66,1.15)	0.32	1.38	(0.96,1.99)	0.08	1.30	(0.90,1.88)	0.16
**Pulmonary thromboembolism**												
Reference	0.66	(0.45,0.99)	0.04	0.75	(0.50,1.12)	0.16	2.10	(1.32,3.34)	0.002	1.97	(1.23,3.14)	0.005
**Cerebrovascular disease**												
Reference	0.58	(0.55,0.61)	< 0.0001	0.68	(0.65,0.72)	< 0.0001	1.66	(1.56,1.78)	< 0.0001	1.55	(1.45,1.65)	< 0.0001
**Myocardial infarction**												
Reference	0.56	(0.45,0.69)	< 0.0001	0.65	(0.53,0.81)	< 0.0001	1.67	(1.30,2.16)	< 0.0001	1.52	(1.18,1.97)	0.001
**Acute renal failure**												
Reference	0.80	(0.60,1.06)	0.12	0.94	(0.70,1.25)	0.65	2.63	(1.90,3.65)	< 0.0001	2.24	(1.61,3.11)	< 0.0001
**Postoperative delirium**												
Reference	0.27	(0.14,0.52)	< 0.0001	0.31	(0.16,0.59)	0.0004	3.30	(1.86,5.87)	< 0.0001	2.57	(1.44,4.61)	0.002
**Surgical site infection**												
Reference	0.91	(0.73,1.14)	0.42	0.95	(0.76,1.19)	0.64	1.72	(1.29,2.29)	0.0002	1.61	(1.21,2.15)	0.001

*HR* hazard ratio, *CI* confidence interval, reference: age of 60–65.

## Discussion

The range of indications for high tibial osteotomy (HTO) is expanding^1,4^, especially in terms of patient age. There are many studies on the optimal age to perform TKA^27,28^. However, there is no consensus regarding the age limit for HTO. In the present study, data from a nationwide Korean registry were used to compare survival rates and perioperative complications based on age. We included 61,145 patients who underwent HTO and evaluated the revision rate and postoperative complications according to age. The survival rate after HTO was significantly higher in Group A (< 60 years) than other groups (≥ 60 years). Additionally, the incidence of perioperative complications after HTO was higher in patients aged > 65 years than in those aged 60–65 or < 60 years. Therefore, surgeons must carefully consider whether to perform HTO on patients aged ≥ 60 years with medial knee OA. To our knowledge, the present study is the first to evaluate HTO revision rates according to age in a large, validated, nationwide cohort. The number of HTO procedures increased by 6.5-fold during the study period, while the number of patients undergoing the procedure after the age of 65 years increased by 8.2-fold.

One of the most important risk factors for survival after HTO is patient age^9,13,15,16,20,21,25,26,29–32^. Although there is no consensus regarding the age limit for HTO, some recent studies have reported no age restriction for successful outcomes after HTO^7,22,23,33,34^. Kuwashima et al.^23^ reported excellent overall survival rates of HTO (94.4% and 84.6% at 10 and 15 years, respectively). Their study could not find any statistical significant difference in survival rate after HTO between the two groups divided by the age (≥ 65 years or not). Moreover, Ruangsomboon et al.^19^ also reported good survival rate of HTO after 4 year follow-up, and concluded HTO in patient ≥ 60 years had good surgical options for OA with acceptable complication. However, other studies showed higher failure rate in old patients than young patients^12,15,20,25,26,31,35^. Keeenan et al.^15^ evaluated the risk factor using a total of 1576 HTO and reported increased incidence of arthroplasty by 8% in each additional year in age patients (relative risk, 1.08). They recommend that careful consideration should be given to patient age when selecting patients for HTO. Similarly, in the present study, patients aged < 60 years had a significantly lower revision rate after HTO than those aged more than 60 years or > 65 years (4.2% vs. 6.4% or 7.3%). However, there was no difference in the revision rates after HTO between patients aged 60–65 years and those aged > 65 years. The total number of revisions 10 years after HTO was significantly lower in patients aged < 60 years than in those aged 60–65 or > 65 year (9.5 vs. 17.8 or 19.1 per 1000 person-years). After adjusting for potential confounders, the revision rate was significantly lower in patients aged < 60 years than in those ≥ 60 years old. Based on our findings, relatively low survival rates of HTO in elderly patients should be noted, especially in patients over 60 years of age.

There are some debate regarding gender effect on survival after HTO^9,15,16,32^, Bouguennec et al.^9^ reported poor survival in male patients after HTO. However, other studies showed poor survivals in female patients^16,32^. Pannel et al.^32^ reported 1.38 increased risk of conversion to arthroplasty in female patients compared with male patients. Similarly to their results, we found poor survival in female patients in all age groups at 5 and 10 years after HTO.

With regard to perioperative complications after HTO, to our knowledge, the significant adverse outcomes associated with patient age have not been investigated thus far. In the present study, the preoperative incidence of underlying medical diseases was significantly higher in patients aged 60–65 and > 65 years than in those aged < 60 years. In particular, the CCI score was significantly higher in patients aged ≥ 60 years than in those aged < 60 years. As expected, perioperative complications such as CVA, MI, and delirium were significantly less common in patients aged < 60 years than in those aged 60–65 years after adjusting for confounders. Moreover, patients aged > 65 years also had higher incidences of PTE, CVA, MI, ARF, and delirium than those aged 60–65 years. In terms of TKA and UKA, similarly, the results were worse as the patient's age increased. Klasan et al.^36^ reported that Post-TKA mortality rate was higher in the elderly group and general complication rate was also higher in the elderly group. Lee et al.^27^ revealed that the mortality tended to increase with age. Kennedy et al.^37^ grouped the patients by age at UKA (< 55, 55 to < 65, 65 to < 75, 75 +) and the revision rate was not higher in youger patients. Otherwise, the clinical scores had more significant improvement in youger group. Therefore, surgeons should discuss possible perioperative complications with patients or their families before performing HTO.

The strength of the present study is that it used one of the largest datasets (NHIS) worldwide. In Korea, health insurance is mandated by law and covers up to 99% of the population. Using a well-designed statistical technique and multivariate regression analysis, we reduced confounders. In addition, we stratified patients into three age groups (< 60 years, 60–65 years, and > 65 years).

However, the present study has several limitations. First, claims-based studies have inherent problems—diagnostic and procedure codes can fail to reflect a patient’s actual medical history. Second, detailed clinical information of individual patients was not available. Thus, the clinical and functional outcomes could not be compared based on age. Third, in study design, it may be more meaningful to divide the age group of the patient into more than three groups or to find the age that is a cut-off value. However, it was our best option to divide the age groups into three groups which had a relatively high number of surgeries. It was not simple comparison between younger and older patients, and could be more detailed comparison between different age groups. Finally, there were many confounding factors even after considerable adjustment. For example, the severity and extent of knee OA are can be identified radiologically, and therefore, it was not considered in the present study. And many other confounders such as degree of deformity, body mass index and status of activity were not considered in the analysis. Despite these limitations, to our knowledge, the current study is the first large-scale, long-term, well-designed cohort study to evaluate the effectiveness of HTO according to age. Nevertheless, a randomized, level 1 study is required to compare these two well-documented surgical methods used to treat unicompartmental knee OA.

## Conclusions

The HTO can delay conversion to TKA and can be used as a definitive treatment for knee OA, especially in young patients (aged < 60 years) without serious perioperative complications. And the survival rate was found to be inferior in old patients (aged ≥ 60 years) and more perioperative complications than young patients. Therefore, the patient age is one of the predicting factors for a high risk of failure after HTO.

## Data Availability

Korean National Health Insurance Claims Data are available on reasonable request. Study protocol, statistical code : available from the author JKS (e-mail, seonbell@jnu.ac.kr). Dataset : de-identified datasets generated and analysed during the present study will be made available by request from the Health Insurance & Assessment Service of Korea at https://opendata.hira.or.kr/. After user approaval by the Health Insurance Review and Assessment Service, a remote analysis system (https://ras.hira.or.kr) can be used by receiving a virtualized ID.
